# A Prospective, Multicenter Study Investigating the Effectiveness of an Oscillating Positive Expiratory Pressure (OPEP) Device in COPD Patients in Québec, Canada

**DOI:** 10.1155/carj/2033683

**Published:** 2026-06-08

**Authors:** Claude Poirier, Pierre-Alexandre Ménard, Romina Tate Gemelli, Marie-Claude Dupont, Sheila Wang, Jason Suggett

**Affiliations:** ^1^ Department of Medicine, Centre Inspir’er, Montréal, Québec, Canada; ^2^ Centre D’investigation Clinique de La Mauricie, Trois-Rivières, Québec, Canada; ^3^ Association Pulmonaire Du Québec, Montréal, Québec, Canada; ^4^ GMF Clinique Médicale Mascouche, Mascouche, Québec, Canada; ^5^ Medical & Scientific Affairs, Trudell Medical International, London, Ontario, Canada

## Abstract

**Rationale:**

Clinical benefits of oscillating positive expiratory pressure (OPEP) devices have been previously demonstrated in many respiratory diseases, including chronic obstructive pulmonary disease (COPD). This study aimed to provide patient‐reported outcomes of OPEP treatment in COPD patients in Québec, Canada.

**Method:**

Recruitment took place across 4 sites. Included patients had a COPD Assessment Test (CAT) score greater than 10 and a mucus score of 2 or greater. Aerobika^∗^ OPEP (TMI) devices were provided, with instructions on usage and technique. CAT assessments were collected at baseline and 6 and 12 weeks. At the final visit, patients were asked whether they would continue to use the device and provided ratings on usability and satisfaction.

**Result:**

Data were analyzed from 46 patients. The average CAT score at 6 weeks was significantly lower than baseline (19.02 ± 6.59 vs. 23.65 ± 6.44, *p* < 0.00001), with 31/44 patients (70.5%) reaching the minimum clinically important difference (MCID). Scores decreased further at 12 weeks (16.15 ± 6.06), a significant improvement from 6 weeks (*p* = 0.0052). MCID was reached by 39/44 patients (84.8%) after 12 weeks of treatment. Average 6 and 12 week mucus scores (2.59 ± 1.02 and 2.28 ± 1.13, respectively) were both significantly lower than baseline (3.63 ± 0.97; *p* < 0.00001 at 6 and 12 weeks). Regarding device feedback, 43/44 patients (97.7%) would continue using the device. The proportion of patients that provided 4/5 or 5/5 satisfaction ratings was 40/45 (88.9%), 43/45 (95.6%), and 40/45 (88.9%) for ease of use, quality, and overall satisfaction, respectively. No device‐related adverse events were reported.

**Conclusion:**

Statistically significant clinical improvements were observed with OPEP therapy. This study supports the use of the Aerobika^∗^ OPEP in conjunction with standard of care in COPD patients to manage symptoms and enhance quality of life. Patient feedback shows high levels of acceptance and perceived value of the device.

## 1. Introduction

Chronic obstructive pulmonary disease (COPD) is characterized by airflow limitation in the lungs due to chronic inflammation from irritant gases and particles. This in turn can result in cilia impairment and mucus hypersecretion. Exaggerated sputum production is a hallmark indicator associated with exacerbations, reduced quality of life (QOL), and increased risk of mortality [1–4]. As such, airway clearance is an important solution for respiratory symptom management. A number of pharmacological treatments exist to target various pathophysiologic mechanisms that result in excess mucus in the airways. These may include expectorants or mucolytics, anticholinergics, and bronchodilators [5]. Despite such options, excess trapped mucus still contributes to poor QOL, and patients face higher safety risks than a nonpharmacological alternative [6]. As such, nonpharmacological therapies such as physical rehabilitation, autogenic drainage, and mechanical devices such as high‐frequency chest wall oscillation are also prescribed to facilitate mucus clearance [7, 8]. Indeed, clinical evidence exists to suggest responses to bronchodilators may be enhanced with mechanical mucus clearance devices in stable COPD patients [9].

Oscillating positive expiratory pressure (OPEP) devices are used as a nonpharmacological airway clearance technique (ACT). Positive pressure generated when exhaling against the device’s resistance allows for airway opening, and for air to get behind the mucus. Simultaneously, the oscillations (pressure pulses) create vibrations in the airways, which help to both shake loose thick and sticky mucus, and free cilia. These mechanisms aid in transporting mucus from smaller to central airways, ready to be coughed out.

Clinical benefits of the Aerobika (Trudell Medical International, London, ON, Canada) OPEP device have been previously studied, with improvements in lung function, exacerbation rates, inpatient admission, and physical capabilities in COPD patients [10–13]. Aerobika^∗^ OPEP patients were propensity score matched to both controls or an OPEP alternative, with results showing Aerobika^∗^ users had fewer exacerbations and hospital visits [10, 11]. Additional study results support improved forced vital capacity (FVC), and 6‐min walk distance (6MWD) post‐Aerobika^∗^ OPEP therapy [12, 13]. However, there is growing recognition on understanding the impact of such devices on patient lives, from their perspectives. This study evaluated the impact of Aerobika^∗^ OPEP on QOL outcomes in COPD patients, as measured by the COPD Assessment Test (CAT) over a 12‐week period.

## 2. Materials and Methods

This was a prospective, interventional, multicenter study. Recruitment on a voluntary basis took place across 4 sites in Québec: Centre Inspir’er, Centre d’investigation clinique de la Mauricie, GMF Clinique médicale Mascouche, and Centre médical St‐François. Patients were included if they met the following criteria: diagnosed with COPD, a baseline CAT score greater than 10, a baseline mucus score of 2 or greater for item 2 of the CAT pertaining to amount of mucus felt in the chest, and cognitive ability to use the device. Patients were excluded if they were currently using or had used a positive expiratory pressure (PEP) or OPEP device in the past 12 months.

All recruited patients were provided with an Aerobika^∗^ OPEP device for the duration of the study and instructed on usage and technique. Patients were advised to complete at least 2 sessions daily, with each session being 10–20 min in duration. Patients were also advised about potential side effects.

The CAT was administered as a QOL assessment. The CAT is a validated, 8‐item questionnaire used to assess the degree of which COPD impacts daily life and wellbeing. Scores range from 0 to 40, with higher scores indicating a greater impact. Scores are categorized in 4 groups: low (< 10), medium (10–20), high (21–30), and very high (31–40). Recognized improvement was determined with the minimum clinically important difference (MCID), where threshold has been previously established to be two points [14]. Patients completed the CAT at their initial visit (Week 0) and again at their Week 6 and Week 12 follow‐up visits. In addition, a qualitative feedback questionnaire regarding the Aerobika^∗^ OPEP device was administered at the 12‐week follow‐up.

Pre‐ and post‐OPEP measurements were compared with paired *t*‐tests, with an alpha level set at 0.05. QOL scores were reported as average ± standard deviation, and categorical results were expressed as proportions and percentages.

This study was conducted in accordance with all applicable regulatory requirements and in accordance with laws and regulations on personal health information, good clinical practice, and all applicable patient confidentiality requirements and the guiding principles of the Declaration of Helsinki. Ethics review was completed by VERITAS IRB Inc, an accredited central IRB based in Kirkland, Québec.

## 3. Results and Discussion

### 3.1. Results

Data from 46 patients across 3 sites were included for analysis. Of the cohort, 30/46 (65.2%) were female. No adverse events related to the Aerobika^∗^ OPEP device were reported throughout the course of treatment.

#### 3.1.1. Quality of Life

**FIGURE 1:**
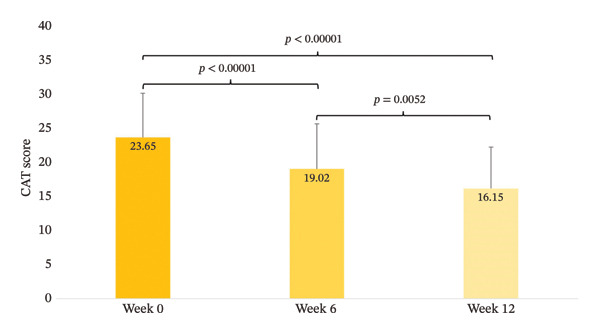
Average CAT scores of COPD patients measured after 0, 6, and 12 weeks of Aerobika^∗^ OPEP treatment.

**FIGURE 2:**
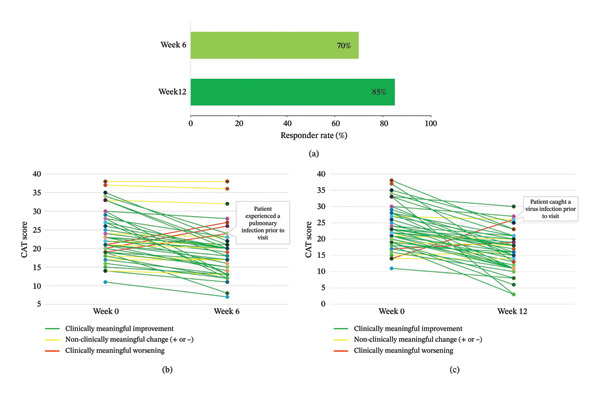
(a) Percentage of patients that reached MCID with the CAT after 6 and 12 weeks of Aerobika^∗^ OPEP treatment. (b) Changes in patients’ CAT scores from baseline (Week 0) to Week 6 of Aerobika^∗^ OPEP treatment. (c) Changes in patients’ CAT scores from baseline (Week 0) to Week 12 of Aerobika^∗^ OPEP treatment.

The average CAT score after 6 weeks of OPEP intervention was significantly lower than baseline visit (19.02 ± 6.59 vs. 23.65 ± 6.44, *p* < 0.00001; Figure 1), with a difference of 4.55 ± 5.29 points. After 6 weeks, 31/44 patients reached MCID improvements in QOL, demonstrating a responder rate of 70.5% (Figure 2a). Of the remaining 13 patients that did not reach MCID, 10 (22.7%) showed no change or a change of ±1 in their scoring and 3 (6.8%) scored higher than 2 points compared with their baseline visit score (Figure 2b). One of such patients experienced a pulmonary infection prior to their visit.

Following 12 weeks of OPEP use, the average CAT score (16.15 ± 6.06) decreased by 29% from baseline (*p* < 0.00001), with an average difference of 7.50 ± 6.94 points. This average CAT score was also significantly lower when compared with 6‐week results (*p* = 0.0052) (Figure 1), with the average difference score being 2.04 ± 7.61 points lower. At 12 weeks, 39/46 patients reached MCID improvements in QOL, demonstrating a responder rate of 84.8%, a 20.2% increase from Week 6 results (Figure 2a). Of the remaining 7 patients, 5 (10.9%) showed no change or a change of ±1 in their scoring and 2 patients (4.3%) scored two or more points higher than their baseline visit score (Figure 2c). One of such patients experienced a viral infection prior to their visit.

**FIGURE 3:**
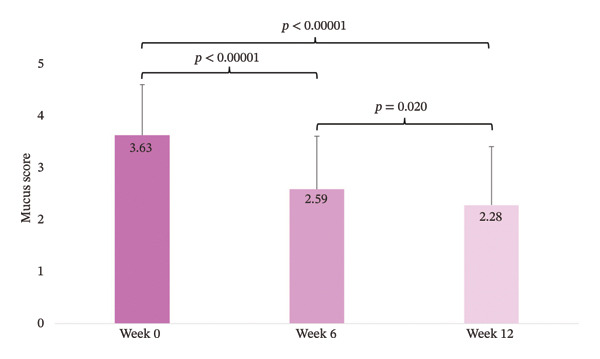
Average mucus scores of COPD patients measured after 0, 6, and 12 weeks of Aerobika^∗^ OPEP treatment.

Average mucus scores from the CAT were reported at 6 and 12 weeks (2.59 ± 1.02 and 2.28 ± 1.13, respectively). Both were significantly lower than the average baseline score (3.63 ± 0.97; *p* < 0.00001 at 6 weeks, *p* < 0.00001 at 12 weeks). Statistical significance was also reached between the 6‐ and 12‐week timepoints (*p* = 0.020) (Figure 3). Mucus scores were shown to be reduced by 35% by 12 weeks.

**FIGURE 4:**
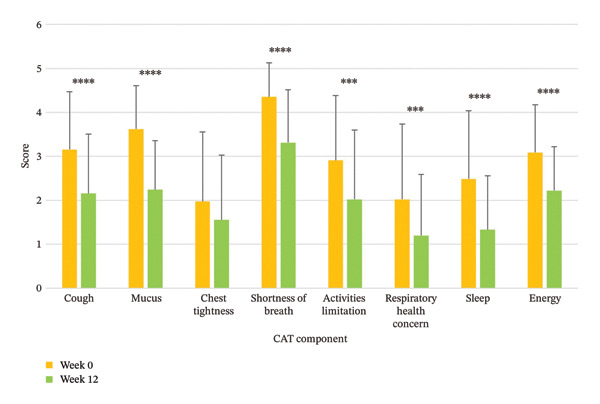
Average scores of each CAT item of COPD patients measured at Weeks 0 and 12 of Aerobika^∗^ OPEP treatment. ^∗∗∗^ *p* < 0.001; ^∗∗∗∗^ *p* < 0.0001.

Each component of the CAT was also analyzed between baseline and Week 12 results. Significant reduction in scores was observed for all components, with the exception of chest tightness (Figure 4). Score changes between the two timepoints are as follows for each component: cough (3.16 ± 1.31 and 2.16 ± 1.35; *p* < 0.00005), mucus (reported previously), chest tightness (1.98 ± 1.57 and 1.56 ± 1.47; *p* = 0.07), shortness of breath (4.36 ± 0.77 and 3.31 ± 1.20; *p* < 0.00001), activities limitation (2.91 ± 1.47 and 2.02 ± 1.57; *p* = 0.0004), respiratory health concern (2.02 ± 1.71 and 1.20 ± 1.39; *p* < 0.0004), sleep (2.49 ± 1.55 and 1.33 ± 1.22; *p* < 0.0001), and energy (3.09 ± 1.08 and 2.22 ± 1.00; *p* < 0.0001).

#### 3.1.2. Qualitative Feedback

**FIGURE 5:**
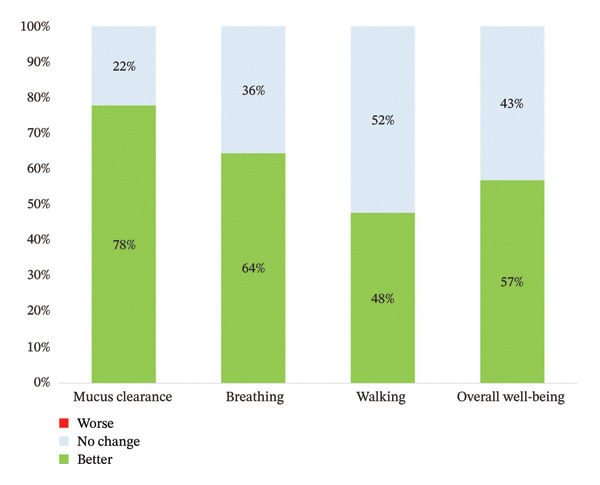
Stacked graph depicting the percentage of patients that responded feeling better, no change, or worse, in their ability to clear mucus, ability to breathe, ability to walk, and overall well‐being. Responses were based on 12 weeks of Aerobika^∗^ OPEP use.

At their 12‐week follow‐up, patients were asked whether they felt better (3 points), no change (2 points), or worse (1 point) about symptom management, namely, mucus clearance, breathing, walking, and overall wellbeing. The average score for mucus clearance was 2.78 ± 0.42, with 35/45 (77.8%) of the patients reporting their clearance has improved and 10/45 (22.2%) citing no change. The average breathing score was 2.64 ± 0.48, with 29/45 (64.4%) perceiving an improvement, while 16/45 (35.6%) did not notice any changes. Average walking score was 2.48 ± 0.50, with 21/44 (47.7%) feeling better about their ability, and 23/44 (52.3%) reported their ability remaining the same before OPEP use. Finally, the score for overall wellbeing was 2.57 ± 0.50, with 25/44 (56.8%) noting an improvement and the remaining 19/44 (43.2%) no change (Figure 5). No patients reported feeling worse for any of the four items.

**FIGURE 6:**
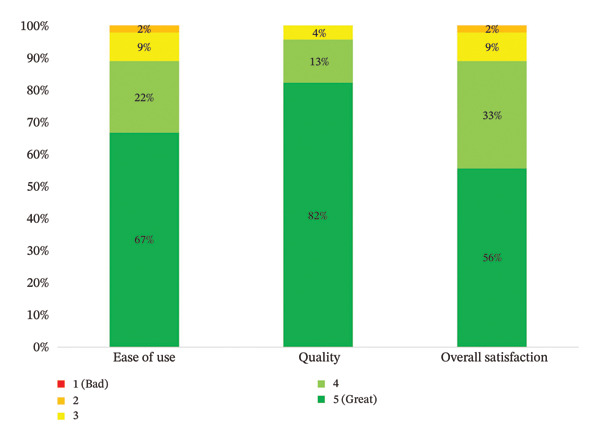
Proportions of patients ranking aspects of the Aerobika^∗^ OPEP device from 1 (bad) to 5 (great), after 12 weeks of usage.

With respect to device‐specific feedback, 43/44 (97.7%) patients would continue to use the Aerobika^∗^ OPEP device. Patients also answered on a scale from 1 (*bad*) to 5 (*great*) the following on the device: ease of use, quality of device, and overall satisfaction. Average scores and proportion of patients that responded with a 5 rating for each aspect were as follows: ease of use: 4.53 ± 0.76, 30/45 (66.7%); quality of device: 4.78 ± 0.52, 37/45 (82.2%); and overall satisfaction: 4.42 ± 0.75, 25/45 (55.6%). No patients rated the device a 1 for any of the items. Figure 6 provides a breakdown of patient scoring.

**FIGURE 7:**
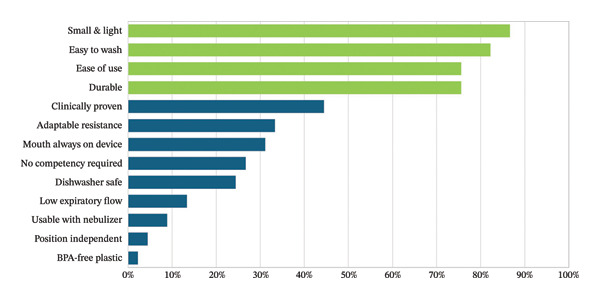
Percentages of patients that selected presented features of the Aerobika^∗^ OPEP device as important to have in an OPEP device.

Finally, patients selected what features of the Aerobika^∗^ OPEP device they perceived as most important (Figure 7). Notably, 39/45 (87%) highlighted “lightweight and small,” while 37/45 (82%) also highlighted “easy to take apart and clean.” High importance was also placed on “ease of use” and “durability,” both with 34/45 (76%) of patients in agreement.

## 4. Discussion

This study evaluated the effect of daily Aerobika^∗^ OPEP device use on health status evaluated by CAT in COPD patients. The results demonstrated that use of the device improved day to day symptoms, with both statistical and clinical significant improvements demonstrated in overall CAT scores at both 6 and 12 weeks of OPEP use. Approximately 70% of the patients in the study showed clinically significant improvements in their QOL after 6 weeks OPEP therapy, and this increased to approximately 85% at the 12‐week point. These findings are consistent with that of previous Aerobika^∗^ OPEP studies in COPD patients, with posttreatment timepoints ranging from 42 days to 24 weeks. In these studies, not only was a reduction in total CAT score observed at all follow‐ups [12, 13, 15–17], clinical responses across studies also ranged from 62% to 82.5% [15, 17]. Positive results were also observed with spirometry and impulse oscillometry (IOS), and improvements were sustained at 24 weeks [12, 13]. It can be inferred that these collective results were achieved through the OPEP device facilitating airway opening and airway clearance.

This study shows that a large majority of patients received meaningful benefits from the device, with consistent usage resulting in improved symptoms and less disruptions in day‐to‐day activities. It is worth noting that none of the patients had any changes to their prescribed medication throughout their participation. In addition, the change in the average CAT total score from 23.65 at baseline to 19.02 and 16.15 at 6 and 12 weeks, respectively, shifts COPD impact from the high to medium category. While recognizing that this is the average, and not individual, score, such a shift shows that this nonpharmacological intervention can significantly improve patient quality of life and has the potential, therefore, to reduce the reliance on pharmacological management plans.

With regards to qualitative feedback, most patients perceived a positive change in their ability to clear mucus, breathing, and overall wellbeing. The largest impact was the ability to clear mucus, with 78% patients noting their ability was better after device use. In keeping with these results, decreased scores were found in the CAT item inquiring about the amount of mucus in the chest. These findings in parallel support the effectiveness of the Aerobika^∗^ OPEP device’s oscillatory feature in loosening excess mucus in the lungs. Patients also provided high scores on aspects of device usage and general satisfaction. It is worth noting that patients selected ease of use and durability as some of the most important characteristics they look for in an OPEP device. At the same time, 89% and 95% of the patients provided favorable feedback on ease of use and quality of the Aerobika^∗^ OPEP device, respectively. These features being met for the majority of patients may have been influential in 97.7% wanting to continue using the device. High levels of patient satisfaction with Aerobika^∗^ OPEP have previously been reported; Harkness et al. analyzed 812 survey responses and found 96% of the patients found it easy to use, 97% would continue to use, and 94% overall satisfaction [18]. Real‐world positive reception to the device may reflect high rates of therapy adherence, and thus sustained, improved outcomes. Consistent use of the device may be further supported by the fact that no significant side effects were reported in the study. Despite collectively positive feedback, no definitive remarks can be made to adherence as the study did not include a method of continuously or intermittently monitoring device usage. As such, it is unclear whether relationships exist between adherence levels and outcomes.

A couple of limitations should be acknowledged. While recruitment took place across 4 sites, the sample size analyzed is small. Additionally, there was no control arm and, therefore, not a randomized controlled trial. It would be of interest in future research to not only study larger populations but also under a randomized design for a robust comparison. It is also worth noting that by administration of the CAT as the only QOL measure, results are based on each individual’s perception, which can vary. However, the CAT is a validated instrument that can predict disease impact and severity via pulmonary function testing (PFT) [19–21], and the primary objective of this study was to gather data from the patients’ perspective.

## 5. Conclusion

The findings of this study provide strong evidence supporting the use of the Aerobika^∗^ OPEP device in conjunction with standard of care drug therapy in COPD patients to manage symptoms and enhance QOL. In addition to showing clinically significant benefits in QOL for 85% of the patients studied, the device was found to be easy to use and clean, with 98% of the patients wishing to continue using it. Additional research is needed to look at adherence and impact on patient lives over a longer period of use.

## Author Contributions

All authors contributed to data analysis and manuscript writing. Claude Poirier, Pierre‐Alexandre Ménard, Romina Tate Gemelli, and Marie‐Claude Dupont contributed to study execution. Claude Poirier, Pierre‐Alexandre Ménard, Romina Tate Gemelli, Marie‐Claude Dupont, and Jason Suggett contributed to study design. Claude Poirier was involved in ethics submission to VERITAS IRB.

## Funding

This study was supported by Trudell Medical International.

## Conflicts of Interest

Authors Sheila Wang and Jason Suggett are employees of Trudell Medical International within the medical and scientific affairs department. The remaining authors declare no conflicts of interest.

## Data Availability

Research data are not shared.
